# Mitigating the Negative Effects of Internet Browsing on Young People’s Resilience and Outlook on Life Through Classic Grimms' Fairy Tales: Exploratory Randomized Controlled Study

**DOI:** 10.2196/76770

**Published:** 2025-08-06

**Authors:** Congcong Hou, Thomas Foscht, Barbara Duffek, Annisa Arigayota, Andreas Benedikt Eisingerich

**Affiliations:** 1 Faculty of Commerce Kyushu Sangyo University Fukuoka Japan; 2 Department of Marketing University of Graz Graz Austria; 3 Robinson College of Business Georgia State University Atlanta, GA United States; 4 Imperial College Business School Imperial College London London United Kingdom

**Keywords:** internet browsing, classical fairy tales, Brothers Grimm, resilience, outlook on life, randomized controlled study

## Abstract

**Background:**

Internet browsing is a daily activity for many young people. However, how internet browsing affects young people’s resilience and positive (vs negative) outlook on life remains largely unaddressed. Critically, how reading classical fairy tales may help mitigate the influence of internet browsing on resilience and foster a more positive rather than negative outlook on life has yet to be explored.

**Objective:**

This study examines the influence of internet browsing on young people’s resilience and positive (vs negative) outlook on life. Furthermore, this study aims to examine the potential mitigating effect of reading classical Grimms' fairy tales, such as Hansel and Gretel and Little Red Riding Hood, on the relationship between internet browsing and postgraduate students’ resilience and outlook on life.

**Methods:**

A randomized controlled study was conducted using a 2 (internet browsing vs no internet browsing) × 2 (reading a classical fairy tale vs no classical fairy tale) between-subjects design. All study participants (N=412) were postgraduate students and randomly assigned to one of the study’s 4 conditions and answered a brief questionnaire, examining their resilience and positive versus negative outlook on life. To examine the potential mitigating effect of classical fairy tales on the relationship between internet browsing and resilience as well as positive versus negative outlook on life, we conducted an exploratory bootstrapping-based moderated mediation analysis with 5000 resamples.

**Results:**

The results showed a significant moderating role of reading classical Grimms' fairy tales on the negative effect of internet browsing on postgraduate students’ resilience and outlook on life. Specifically, when study participants browsed the internet, they reported a more positive outlook on life when they read a Grimms' fairy tale (read fairy tale: mean 5.46, SD 0.151 vs not read fairy tale: mean 3.01, SD 0.150, SE 0.213, 95% CI –2.860 to –2.024; *P*<.001). Furthermore, the results showed that when participants browsed the internet, they indicated significantly greater resilience when they read a Grimms' tale (mean 4.62, SE 0.179, 95% CI 4.271-4.976) than when they did not (mean 2.59, SE 0.179, 95% CI 2.243-2.945). In addition, an exploratory analysis demonstrated that the effect of internet browsing on outlook on life is mediated by resilience (effect 0.85, SE 0.17, 95% CI 0.52-1.20).

**Conclusions:**

The findings of this study show that reading a classical Grimms' fairy tale, such as Hansel and Gretel or Little Red Riding Hood, helped mitigate the negative effects of internet browsing on postgraduate students’ resilience and outlook on life.

**Trial Registration:**

ISRCTN 16972408; https://www.isrctn.com/ISRCTN16972408

## Introduction

### Overview on Internet Browsing, Young People’s Resilience, and Outlook on Life

Internet browsing has the potential to be informative and enriching, but unfiltered use can also have negative psychological effects, especially on younger users who are still developing their sense of self and resilience. Young people who frequently browse news feeds and social media can be inundated with distressing headlines, crisis updates, and alarming videos [1]. This continuous exposure to negative events, often without the buffer of context or constructive discussion, can fuel anxiety and pessimism about the future. Moreover, various internet platforms often showcase carefully curated highlight reels, which can lead to unrealistic expectations and self-comparison [2]. Constantly measuring one’s life against seemingly “perfect” snapshots can erode self-esteem and foster a negative self-image. That is, being bombarded with negative or unrealistic messages can create a sense that the world is filled with endless threats and personal inadequacies.

Numerous platforms on the internet, including social media, are strongly addictive, and young people may find themselves hooked [3,4]. Relying heavily on online communication can also limit meaningful face-to-face interactions, as in-person encounters often require young people to practice social skills such as reading body language, managing conflicts, and empathizing with peers. Furthermore, the internet can provide a form of instant escape [5,6], browsing videos, memes, or messaging apps, whenever stress or discomfort arises. Resilience, however, develops partly through experiencing and overcoming manageable stressors, such as small disagreements with friends or navigating unfamiliar situations. If young people rarely face these real-life challenges, or if they continuously escape them through digital means, they may struggle to build strong coping mechanisms.

Moreover, social media platforms as well as search engines and even various news sites often use algorithms that personalize content [7-9]. If a user frequently engages with negative, sensational, or critical information, the algorithm may feed them more of the same, creating a feedback loop of negativity [10]. People also naturally gravitate toward content that reaffirms their existing beliefs and feelings [11]. A young person feeling discouraged may seek out material that confirms a pessimistic worldview, reinforcing a negative outlook and limiting exposure to hopeful or solution-oriented perspectives. When the online environment consistently mirrors or amplifies negative thoughts, it can be difficult for young people to see alternative viewpoints or foster optimism [12,13]. This echo chamber effect can cement a sense of fatalism and hopelessness [14].

The internet provides an endless stream of entertainment and quick answers. As a result, some young users may become accustomed to immediate gratification [15,16]. This can weaken patience, perseverance, and the ability to tolerate frustration when tasks become challenging. Rapid scrolling through social media feeds, video clips, shorts, or headlines can condition the brain to seek quick hits of novelty and pleasure. This makes it harder to stay focused on sustained tasks, such as reading a book, thinking through a difficult problem, or engaging in a complex real-world project.

Resilience often involves the ability to concentrate on long-term goals and persevere despite difficulties. When young people are conditioned to expect instant results or quick solutions, they may be more prone to giving up in the face of real-world hurdles. In addition, the anonymity of the internet can embolden bullying behavior [17,18]. Young people who are targeted may experience heightened stress, anxiety, and feelings of isolation, which can diminish their ability to cope with adversity. Seeing peers’ social activities in real time can also lead to feelings of exclusion or jealousy if they are not included [19-21]. This ongoing sense of missing out can erode self-esteem and overall life satisfaction. A vital component of resilience is a supportive social network. When online spaces become sources of bullying or pressure, it undermines that support and can negatively affect a young person’s self-confidence and emotional well-being [22-25].

In addition, internet browsing may foster a largely passive role, scrolling through feeds, or watching videos, leading to fewer opportunities for skill-building or tangible accomplishments. The upshot is that many forms of digital engagement encourage consumption over creation. Young people may spend less time engaging in problem-solving, artistic pursuits, or physical activities that build self-efficacy and pride in achievement [26-28]. In this research, we define resilience as the capacity to adapt positively to adversity, major changes, or setbacks by managing emotional responses, maintaining persistence, and viewing challenges as opportunities for growth. Outlook on life is defined as a future-oriented psychological disposition characterized by positive anticipation, hope, and confidence that life events will unfold favorably. Thus, as part of this research, we aimed to answer the following research questions: To what extent, if at all, does internet browsing affect young people’s self-expressed resilience and outlook (positive vs negative) on life. Furthermore, we aimed to answer how reading a classical Grimms' fairy tale may help mitigate the impact of internet browsing on young people’s resilience and outlook on life. We discuss the potential influence of classical fairy tales in the next section.

### Overview on Grimms' Classical Fairy Tales, Young People’s Resilience, and Outlook on Life

Classical Brothers Grimm fairy tales, such as Hansel and Gretel or Little Red Riding Hood, have endured for centuries precisely because they speak to fundamental human experiences of challenge, danger, and ultimate survival [29]. Grimms' fairy tales often introduce universal anxieties (fear of abandonment, danger from strangers, isolation in the woods) in a way that is contained within a story [30]. Young and older people who engage with these tales can process and explore fears vicariously [31,32]. Because the stories are set in a fantastical or once-upon-a-time realm, it becomes psychologically safer to confront these themes [33-35].

Furthermore, Grimms' classical fairy tales can affirm moral values such as bravery, kindness, and clever thinking in the face of wrongdoing. Hansel and Gretel teaches the importance of sticking together and helping one another, while Little Red Riding Hood underscores the risks of straying from the path or trusting too easily. These lessons can instill a sense of moral clarity that contributes to confidence and resilience in real-life choices [36]. Despite their dark elements, many classic tales promise hope in the form of a “happy ending” [37]. This element reassures readers that hardships, no matter how dire, can be resolved and that it is possible to emerge from difficult times intact and, indeed, stronger. As young people read or listen to these stories, they learn the language of emotions: fear, relief, joy, sadness, and hope. Building an emotional vocabulary helps them understand and express their own feelings, which is a cornerstone of emotional resilience [38,39].

### Background on The Moderating Effect of Grimms' Fairy Tales on the Influence of Internet Browsing on Resilience and Outlook on Life

Internet browsing has become an integral part of daily life, especially for young people who have grown up with digital technology. Although it offers numerous benefits such as access to information, there are ways in which it might weaken resilience and contribute to a more negative outlook on life [40]. For instance, the internet provides an unending stream of news, much of which can be negative, focusing on crises, disasters, and conflicts [41-43]. Continuous exposure to such content can lead to anxiety, desensitization to violence, and a worldview that emphasizes the negative aspects of life especially for younger people [42-44]. Moreover, online interactions can expose young people to bullying [45,46], which can be more pervasive and relentless than traditional forms due to the anonymity and reach of the internet. This can significantly impact self-esteem, increase anxiety, and decrease resilience.

Platforms often showcase idealized or curated versions of others’ lives, leading to unrealistic comparisons [47]. Young people might feel inadequate or that their lives are less fulfilling, which can foster envy, depression, and a negative self-image, undermining personal resilience [25,40,48]. In addition to this, the constant exposure to others’ experiences can make individuals feel they are missing out on life, leading to dissatisfaction with their own lives and a diminished capacity to find contentment in everyday moments [49,50]. Moreover, the internet is a vast repository of information, which can lead to cognitive overload. This constant switching between tasks or pieces of information can reduce the ability to focus on and solve complex problems, potentially weakening resilience when faced with real-life challenges.

There may also be the temptation for and a tendency toward shallow browsing rather than deep learning or engagement [51,52], which might not foster the deep thinking or critical analysis necessary for building resilience through overcoming long-term or complex issues. Although the internet connects people globally, it can also lead to physical isolation, reducing face-to-face interactions that are crucial for developing social skills, empathy, and support networks that are foundational for resilience [52,53]. The nuances of human interaction, like reading body language or dealing with conflict in person, might be less practiced, leading to difficulties in handling real-life social complexities. The internet also frequently provides instant access to information, entertainment, and social validation, which can skew perceptions of effort versus reward in real life. This might lead to impatience or a lack of perseverance when faced with tasks that require sustained effort, thus affecting resilience [39].

Although classical Grimms' fairy tales include dark or frightening elements, these stories offer powerful lessons in overcoming adversity. For instance, Grimms' tales are known for their darker undertones, showing children in perilous situations. However, these narratives almost always culminate in liberation or redemption, conveying that hope can persist even when circumstances seem dire. Seeing peers (albeit fictional) succeed independently fosters the belief that one is capable of self-reliance and problem-solving in moments of difficulty. This narrative structure underlines that, no matter how overwhelming a problem appears, there is a path to resolution, fostering optimism and fortitude in readers. In doing so, fairy tales allow children to experience fear, tension, and relief in a controlled, fictional context. They learn that it is possible to feel frightened but still move forward, a concept vital to developing emotional regulation skills.

Furthermore, traditional fairy tales tend to have clear moral lines. Acts of kindness, bravery, or cleverness bring the protagonists closer to safety; malicious or deceitful behavior is penalized. These straightforward moral frameworks help children internalize the belief that virtuous behavior is both valued and effective. Though often overshadowed by more dramatic elements, moments of kindness or cooperation (eg, siblings helping one another) highlight the importance of empathy. Recognizing this promotes healthy social-emotional development in children. Young readers see characters who suffer, facing hunger, abandonment, or trickery, and learn that hardship does not define one’s destiny.

Thus, although steeped in dark imagery, the ultimate message of these tales is one of empowerment: Young people can confront formidable challenges and still emerge triumphant. In a world that can sometimes feel overwhelming, reading these classic fairy tales can serve as a reminder that adversity may be part of the journey, but so, too, is the promise of a brighter ending.

Internet browsing can sometimes blur moral lines or leave young people feeling confused or jaded, particularly when exposed to ambiguous news events or negative online behaviors. Fairy tales, by contrast, often highlight moral clarity and the redemptive power of virtue. Classic Grimms' tales are known for containing darker elements than modern retellings. Although this can be unsettling, it also provides a nuanced understanding that the world has dangers, yet these dangers can be navigated. Critically, the fairy tale characters’ mistakes and subsequent growth can teach resilience by showing that errors are part of the learning process, a lesson that can be overshadowed by the instant gratification and perfection often depicted online. These tales typically conclude with a resolution that underscores the possibility of overcoming adversity. This sense of adversity being an integral part of life’s story, and that it can, indeed, be mastered are crucial for developing an “I can handle this” mindset, particularly in a digital era that often leaves negative events unresolved or amplified.

## Methods

### Study Design

The study was registered in ISRCTN. The completed CONSORT-SPI (Consolidated Standards of Reporting Trials statement for social and psychological interventions) 2018 checklist is included in Multimedia Appendix 1. As part of this study, we used a 2 (internet browsing vs no internet browsing) × 2 (reading a classical fairy tale vs no classical fairy tale) between-subjects experimental design. Over the course of 2 weeks in spring 2025, posters and flyers were used to invite postgraduate university students to take part in a study on student well-being and daily activities. Participants were randomly allocated to 1 of the 4 study conditions using a random 1-4 number generator (condition 1: internet browsing + reading a classical fairy tale; condition 2: internet browsing + no classical fairy tale; condition 3: no internet browsing + reading a classical fairy tale; condition 4 [control group]: no internet browsing + no classical fairy tale). The study was conducted in a lab experimental setting on a university campus.

In condition 1, participants were invited to spend 20 minutes to freely browse the internet on any digital device they had with them (eg, smartphone, tablet, laptop). Extra care was taken to inform study participants that they could browse the internet very freely and visit any site they want and that absolutely no data were collected based on which sites they visited. Research assistants were trained and instructed to look at a phone themselves, so that participants did not feel observed during the study time period. After 20 minutes of free internet browsing, participants were invited and given 20 minutes to read either the classical fairy tale Hansel and Gretel or Little Red Riding Hood (the Brothers Grimm version; randomly assigned, so that some participants read Hansel and Gretel and others read the Little Red Riding Hood fairy tale). Finally, study participants completed a brief survey. More specifically, across all 4 conditions, participants answered a brief survey with Likert-scale measurement items (1=strongly disagree, 9=strongly agree) capturing participants’ positive versus negative outlook on life as well as resilience. Detailed measurement items are listed in Table 1. The measurement items to capture resilience were adapted from previously published work [54,55]. We used reverse-coded measurement items as part of our measurement of resilience and outlook to reduce response bias. The measurement items used for this study were also adapted from prior published research [56,57]. Again, reverse-coded measurement items were used as part of the scale to reduce response bias. In addition, in condition 1 and condition 2, participants answered questions about whether they enjoyed browsing the internet and found it interesting as controls. Furthermore, as controls, participants in condition 1 and condition 3 answered questions about whether they enjoyed reading the fairy tale and whether they were familiar with the fairy tale that they had read. Across all 4 conditions, participants were asked whether they could guess the study’s purpose, and none did. In condition 2, participants were invited to spend 20 minutes to freely browse the internet as in condition 1. After browsing the internet, participants in condition 2 completed the brief survey. In condition 3, participants were randomly allocated to either read Hansel and Gretel or Little Red Riding Hood and given 20 minutes to read the fairy tale and subsequently complete a brief survey as in condition 1. Finally, in condition 4 (control group), participants simply completed the brief survey.

**Table 1:** Study measurement items and reliabilities.

Variable and measures		Response, mean (SD)	Response, range	Statistical test result		AVE^a^		CR^b^	
**Positive versus negative outlook on life**					0.93^c^		0.62		0.87
	“I look forward to each new day in my life.”	4.18 (1.85)	1-9						
	“I do not feel hopeful that things in my life will work out for the best.”^d^	4.25 (2.02)	1-9						
	“I find it difficult to believe that things will be better in the future.”^d^	4.17 (2.04)	1-9						
	“I generally feel positive about my future.”	4.16 (1.86)	1-9						
**Resilience**					0.94^c^		0.79		0.94
	“I believe I can handle whatever life throws at me.”	3.51 (2.16)	1-9						
	“I feel I can manage and adapt to major changes or disruptions in my life.”	3.51 (2.09)	1-9						
	“I am discouraged by setbacks and find it difficult to move forward in life.”^d^	3.47 (2.12)	1-9						
	“I view difficulties in life as opportunities to learn and grow stronger.”	3.35 (2.15)	1-9						
**Enjoyment of internet browsing**					0.76^e^		0.86		0.92
	“I very much enjoyed browsing the internet just now.”	3.80 (1.85)	1-9						
	“I found browsing the internet just now to be interesting.”	3.76 (1.88)	1-9						
**Enjoyment of reading the fairy tale**					0.79^e^		0.90		0.95
	“I enjoyed reading the fairy tale.”	4.42 (1.68)	1-9						
	“I did not like the fairy tale very much.”^d^	4.44 (1.75)	1-9						
**Familiarity with the fairy tale read**					0.84^e^		0.90		0.95
	“I am familiar with the fairy tale that I just read.”	3.89 (2.15)	1-9						
	“I know the fairy tale that I just read very well.”	3.74 (2.11)	1-9						

^a^AVE: average variance extracted.

^b^CR: composite reliability.

^c^Cronbach α.

^d^Reverse coded.

^e^Pearson *r*.

Prior to our main study, we conducted a pretest (n=175) that followed the same recruitment procedure as discussed for our main study and included a neutral distraction task for condition 2, condition 3, and condition 4 to test whether the total time spent in the study affected the results. Specifically, for condition 2, after 20 minutes of internet browsing, study participants were provided with sheets with grids of random consonants and vowels and told “for the next twenty minutes, please find and cross out all instances of the letter ‘X’ and ‘Z’ on the pages provided. Feel free to work at a steady pace. This is not a test of skill, so just do your best to stay focused.” Hence, in condition 2, after 20 minutes of internet browsing, study participants completed the letter cancellation task for 20 minutes, followed by the survey, as discussed in the previous section. Unlike tasks such as puzzles, games, or reading neutral text, letter cancellation was chosen as a distraction task as part of the pretest because it is unlikely to affect resilience or outlook and the task is not harmful or frustrating. The pretest results showed that participants’ expressed resilience did not differ between condition 2 that did include the filler task (n=30; mean 2.48) and the one that did not include the filler task (n=28; mean 2.40; t_56_=0.18, *P*=.86). Similarly, the pretest results demonstrated that participants’ outlook did not differ across condition 2 with (mean 2.93) and without (mean 2.91; t_56_=0.05, *P*=.96) the filler task. Moreover, in the pretest of condition 3, after 20 minutes of fairy tale reading, study participants completed the letter cancellation task, followed by the survey. The pretest results demonstrated that resilience did not differ between condition 3 that included the filler task (n=28; mean 3.20) and condition 3 that did not include the filler task (n=29; mean 3.22; t_55_=0.05, *P*=.96). The pretest results also indicated that outlook did not differ across condition 3 with (mean 4.21) and without (mean 4.10; t_55_=0.18, *P*=.86) the filler task. Finally, in the pretest for condition 4, participants completed the letter cancellation task for 40 minutes, followed by the survey. Pretest results showed that participants’ resilience did not differ between condition 4 with (n=30; mean 3.27) and without (n=30; mean 3.38; t_58_=0.22, *P*=.83) the filler task. Further, the pretest showed that participants’ outlook did not differ between condition 4 with (mean 4.08) and without (mean 3.97; t_58_=0.19, *P*=.85) the filler task. Thus, total time spent in the study did not impact the results, and no filler task was used for our main study to help minimize participants’ time in the study. After the pretest, all participants were debriefed about the purpose of the study and about the purpose of letter cancellation as a time-filler.

**Ethical Considerations**

All participants were informed that the data collected will inform academic research, will not be used for other commercial purposes, and will be anonymized and treated with strict confidentiality and that they were free to stop and withdraw from the study at any time and without giving a reason. Participants were informed that the data from participants who stopped early will not be used as part of the study. We received ethical approval from Kyushu Sangyo University’s ethics committee (#2024-0018). Each study participant was thanked and received a US $5 note as a token of gratitude for taking the time to participate in the study. Written consent forms were obtained from all study participants after they were offered an information leaflet and time (minimum of 24 hours) for consideration was allowed.

**Participants and Procedure**

Our study used a 2×2 between-subjects design to examine the effects of internet browsing and fairy tale exposure on outlook and resilience. A total of 412 participants were recruited, exceeding 100 per condition. A sensitivity analysis was conducted using GPower (version 3.1) for a between-subjects ANOVA with 4 groups. With a total sample size of 412, α of .05, and power (1-β) of .80, the study was sufficiently powered, and the design had adequate sensitivity to detect meaningful main and interaction effects [58]. That is, this sample size provided >99% power to detect medium-sized effects (Cohen *f*≥0.25), which aligns with effect sizes observed in prior digital intervention and narrative psychology research (eg, positive psychology interventions: *d*=0.40-0.60). For smaller effects (*f*=0.15), power remained high at 99%. Even very small effects (*f*=0.10) are detectable with 86% power, surpassing conventional power thresholds (≥80%). This ensured robustness for our primary analyses (ANOVA main effects and interactions) while accommodating plausible effect magnitudes in behavioral health contexts. More specifically, a total of 420 university postgraduate students took part in this study. During the data collection process, 8 participants decided to stop and withdraw from the study. The data from the participants who decided to withdraw were not used as part of the study analyses, resulting in an effective total number of 412 participants for the study (condition 1: n=103; condition 2: n=104; condition 3: n=103; condition 4: n=102).

**Data Analysis**

We examined whether items loaded significantly on intended factors with low cross-loadings [59]. As a next step, we examined the estimates for the average variance extracted (AVE), supporting convergent validity [60] (see Table 1). Moreover, we assessed the possibility that measurement errors can vary in magnitude across items and calculated and compared the AVE for all pairs of constructs to the squared correlation between the 2 constructs of interest [61]. The squared correlation between any pair of constructs was not greater than the respective AVE for each of the constructs in the pair, supporting discriminant validity. In addition, Cronbach α were generally high (Table 1). In order to examine the potential mitigating effect of classic Grimms' fairy tales on the relationship between internet browsing and resilience as well as outlook on life, we conducted a bootstrapping-based moderated mediation analysis with 5000 resamples (PROCESS model 8) [62].

## Results

As evident in Table 2, when participants were allowed to browse the internet, they reported a more positive outlook on life if they read Grimms' fairy tales (read fairy tale: mean 5.46, SD 0.151 vs not read fairy tale: mean 0.3.01, SD 0.150, SE 0.213, 95% CI –2.860 to –2.024; *P*<.001). When participants did not browse the internet, this effect disappeared. A detailed comparison of the cell means is presented in Tables 2-4. Furthermore, the results showed that, when participants browsed the internet, they indicated significantly greater resilience when they read a Grimms' fairy tale (mean 4.62, SE 0.179, 95% CI 4.271-4.976) than when they did not (mean 2.59, SE 0.179, 95% CI 2.243-2.945). When participants did not browse the internet, this effect disappeared (read fairy tale: mean 3.37, SE 0.179, 95% CI 3.014-3.719 vs not read fairy tale: mean 3.26, SE 0.180, 95% CI 2.908-3.617).

**Table 2:** Effects of internet browsing and reading a fairy tale on outlook on life: cell means estimates for the dependent variable of outlook on life.

Condition			Mean		SE		95% CI
**Internet browsing: no**							
	Read fairy tale: no	4.11		0.151		3.813-4.408	
	Read fairy tale: yes	4.177		0.151		3.881-4.473	
**Internet browsing: yes**							
	Read fairy tale: no	3.014		0.150		2.720-3.309	
	Read fairy tale: yes	5.456		0.151		5.160-5.753	

**Table 3:** Detailed cell means comparisons for impact of internet browsing and reading a fairy tale on outlook on life.

Condition			Mean difference^a^		SE		*P* value		95% CI
**Internet browsing: no**									
	Read fairy tale: no-yes	–0.067		0.214		.75		–0.487 to 0.353	
**Internet browsing: yes**									
	Read fairy tale: no-yes	–2.442^b^		0.213		<.001		–2.860 to –2.024	
**Read fairy tale: no**									
	Internet browsing: no-yes	1.096^b^		0.213		<.001		0.677 to 1.515	
**Read fairy tale: yes**									
	Internet browsing: no-yes	–1.279^b^		0.213		<.001		–1.698 to –0.860	

^a^Based on estimated marginal means.

^b^The mean difference is significant at the .05 level.

**Table 4:** Univariate test results for influence of internet browsing and reading a fairy tale on outlook on life.

Internet browsing			Sum of squares (*df*)		Mean square		*F* (*df*)			*P* value	
**Internet browsing: no**								0.098 (1, 408)			.75
	Contrast	0.229 (1)		0.229							
	Error	954.807 (408)		2.340							
**Internet browsing: yes**								131.855 (1, 408)			<.001
	Contrast	308.658 (1)		308.568							
	Error	954.807 (408)		2.340							
**Read fairy tale: no**								26.426 (1, 408)			<.001
	Contrast	61.843 (1)		61.842							
	Error	954.807 (408)		2.340							
**Read fairy tale: yes**								36.006 (1, 408)			<.001
	Contrast	84.262 (1)		84.262							
	Error	954.807 (308)		2.340							

To examine the potential moderating effect of Grimms' fairy tales on the relationship between internet browsing and resilience as well as young people’s outlook on life, we conducted a bootstrapping-based moderated mediation analysis with 5000 resamples (PROCESS model 8) [62]. The analysis was conducted with outlook on life as the dependent variable, internet browsing as the independent variable, resilience as the mediator variable, and whether the participant read a fairy tale as the moderator variable. The sample size was 412 participants. Detailed results are presented in Tables 5-7.

**Table 5:** Moderated mediation regression results.

Predictor			B		SE		*t* (*df*)		*P* value		95% CI
**Dependent variable: resilience^a^**											
	Constant	5.75		0.89		6.39 (408)		< .001		3.98 to 7.51	
	Internet browsing	–2.59		0.57		–4.57 (408)		< .001		–3.70 to 1.48	
	Fairy tale	–1.82		0.57		–3.21 (408)		.002		–2.94 to –0.70	
	Internet browsing × fairy tale	1.93		0.36		5.37 (408)		<.001		1.22 to 2.63	
**Dependent variable: outlook on life^b^**											
	Constant	4.98		0.68		7.37 (407)		< .001		3.65 to 6.31	
	Internet browsing	–2.33		0.42		–5.59 (407)		<.001		–2.15 to –1.51	
	Resilience	0.44		0.04		12.39 (407)		< .001		0.37 to 0.51	
	Fairy tale	–1.51		0.41		–3.65 (407)		.003		–2.32 to –0.69	
	Internet browsing × fairy tale	1.53		0.27		5.74 (407)		<.001		1.00 to 2.05	

^a^R=0.38, *R*²=0.14, mean squared error=3.32, *F*_3,408_=22.36, *P*<.001.

^b^R=0.67, *R*²=0.45, mean squared error=1.70, *F*_4,407_=83.89, *P*<.001.

**Table 6:** Conditional effects of internet browsing on outlook on life at levels of fairy tale presence.

Fairy tale presence	Effect	SE	*t* (*df*)	*P* value	95% CI
Read fairy tale: no	–0.80	0.18	–4.37 (407)	<.001	–1.16 to –0.44
Read fairy tale: yes	0.73	0.19	3.87 (407)	<.001	0.36 to 1.09

**Table 7:** Conditional indirect effects of internet browsing on outlook on life via resilience.

Fairy tale presence^a^	Effect	BootSE	95% CI
Read fairy tale: no	–0.29	0.11	–0.52 to –0.08
Read fairy tale: yes	0.55	0.13	0.32 to 0.81

^a^Index of moderated mediation: index=0.85, BootSE=0.1724, 95% CI 0.52 to 1.20.

The regression results in Table 5 show that internet browsing and reading fairy tales enhanced resilience (B=1.93, SE 0.36; t_408_= 5.37, *P*<.001, 95% bootstrap CI 1.22-2.63), which in turn strengthened positive outlook on life (B=0.44, SE 0.04; t_407_=12.39, *P*<.001, 95% CI 0.37-0.51). Furthermore, the results in Table 5 show that the effect of internet browsing on outlook on life is mediated by resilience (effect 0.85, SE 0.17, 95% CI 0.52-1.20). Together, these results highlight the potentially important effect of internet browsing and reading of fairy tales on outlook on life via resilience.

## Discussion

### Principal Findings

This study shows that classical Grimms' fairy tales, such as Hansel and Gretel and Little Red Riding Hood, serve as profound tools for fostering resilience and nurturing a positive outlook on life among young people. Critically, these tales, although often dark and cautionary, embed timeless lessons through their narratives, characters, and symbolic struggles, which helped mitigate the negative effects of internet browsing on young people’s resilience and outlook on life.

For instance, Grimms' tales often feature protagonists confronting dire circumstances. By witnessing characters navigate fear and emerge stronger, readers internalize the idea that hardship is surmountable and perseverance and adaptability can lead to triumph. Moreover, Grimms' fairy tales often follow a “darkness-to-light” arc, where suffering precedes redemption and crises are temporary. Thus, they can instill hope, suggesting that effort can lead to positive outcomes, a mindset critical for maintaining optimism. A happy ending, even if hard-won, reinforces optimism about overcoming life’s trials and encourages readers to approach obstacles with ingenuity rather than despair. Such narratives reinforce the value of resourcefulness, a key component of resilience.

### Comparison With Prior Work

Previous research notes the impact of internet browsing and the role of frequent news feed browsing as well as social media on anxiety and pessimism on life [1,2]. In addition, prior work has indicated the addictive nature of internet browsing and the potential dangers of personalized content and consumption of negative information on the internet [7-11]. In addition, previous research has shown the effects of various online content on young people’s feelings of hopelessness [12,13,40]. Moreover, prior work indicates the role of the internet in providing a constant stream of news about crises, disasters, and violence [41-43], which may have a significant impact on young people’s lives [42-44]. Various social media platforms also showcase idealized and curated versions of others’ lives, which can lead to reduced contentment and satisfaction with one’s own life [47,49,50]. However, critical questions remain about the effect of random internet browsing on resilience and outlook on life of young people. To the best of our knowledge, this is the first study that examines the role of free internet browsing on postgraduate students’ resilience and outlook on life. In addition to this, previous research has noted the role of classical fairy tales in addressing fundamental human experiences of challenge, danger, and survival [29,30], which may help young people with processing their own fears and worries in daily life [31,32,36]. Critical questions remain regarding the potential mitigating role of reading classical fairy tales in helping reduce the negative effects of internet browsing on resilience and outlook on life of young people. Thus, this study offers critical new insights about the impact of random internet browsing on the resilience of young people as well as their outlook (positive vs negative) on life. Furthermore, this study demonstrates the extent to which reading classical fairy tales may help mitigate the negative impact of internet browsing.

### Limitations and Future Research

This study has a number of limitations that offer promising avenues for future research. Although we explored the role of Brothers Grimm fairy tales in mitigating the negative impact of internet browsing, additional research that examines the role of other classical or modern fairy tales is richly deserving. Moreover, we examined the role of internet browsing and reading classical fairy tales in affecting the resilience and outlook on life of postgraduate students. We invite future research to explore the effects of browsing the internet and reading classical fairy tales for study participants other than postgraduate students (eg, younger or older age groups). In addition, we note that, due to the cross-sectional measurement of both outlook and resilience after the manipulation, the causal mediating role of resilience cannot be confidently established. The current test of resilience as a mediator is exploratory in nature, and future research examining the effects of internet browsing and reading classical fairy tales on outlook and resilience over time is richly deserving. Moreover, previous research indicates that, in addition to resilience, other mediators may exist that help explain the effects of internet browsing on someone’s outlook on life [62-67]. For instance, people may use the internet to gain access to inspiring information, helpful and enabling tools, counselling, or humorous content that may lift their mood, as well as connect with family and friends for enhanced feelings of group membership and communion and contributing positively to society [68-79]. Therefore, we invite additional research to examine how young people may use the internet as a tool and force for good and enhanced resilience as well as positive outlook on life. Furthermore, prior work notes the potential for digital solutions to positively affect mental health and well-being [80] but also indicates privacy concerns people may have when using various internet-based tools [81]. Future research shedding further light on these critical issues is richly deserving. In addition, prior work indicates the need for critical reading of some of the fairy tales and their various, more modern versions [82-85]. For instance, Snow White displays surprising strength and bravery when focusing on inner resilience and moral courage rather than mere physical action. Snow White survived years of psychological abuse and servitude under the jealous queen, yet retains her inherent kindness, optimism, and hope. This emotional resilience in the face of cruelty is a profound strength [86]. Moreover, Snow White chose survival in terror. When sentenced to death in the woods, she does not freeze nor despair. Snow White actively flees through a terrifying, dark, unknown forest, which is a genuinely brave act for anyone, especially a young girl (depicted as very young in the original tale). In addition, Snow White basically rebuilt her life from scratch, demonstrating a courage to start anew. Critically, maintaining compassion and hope in a world that has shown her cruelty requires significant inner fortitude and a special kind of bravery. It is the core of her identity that Snow White refuses to let the evil queen destroy. Through this angle, readers may see that she is a strong and resilient character in a deeply psychological way and that there can be a path to finding hope even in very dark times [87]. Sometimes the quietest forms of strength, such as resilience, hope, and kindness in the face of cruelty, are the most profound, yet the easiest to overlook [88].

By balancing caution with courage and individualism with community, these stories cultivate a resilient spirit and a belief in positive outcomes, proving that even the darkest forests can be navigated with perseverance and heart. Grimms' fairy tales are like ancient mirrors. They reflect both people’s deepest fears and their capacity to outgrow them. What is fascinating is that resilience and optimism often bloom not despite the darkness in these tales but because of it. They do not sugarcoat life’s terrors. Instead, they whisper “Yes, the world has witches and wolves...but also breadcrumb trails, cleverness, and kind woodsmen.” That balance feels deeply human and endlessly relevant.

### Summary

This study shows that reading classical Grimms' fairy tales such as Hansel and Gretel and Little Red Riding Hood can help mitigate the negative effect of internet browsing on young people’s resilience and lead to a more positive versus negative outlook in their lives. Classical Grimms' fairy tales are more than escapism; they can be blueprints for resilience and optimism. Through adversity, resourcefulness, and hope, they equip readers with mental frameworks to confront life’s challenges.

Many fairy tales end with hope, villains are defeated, families reunite, and protagonists emerge stronger. This narrative arc fosters optimism and a sense of agency, countering the helplessness or cynicism that can arise from exposure to negative online content (eg, doomscrolling, cyberbullying). Reading fairy tales also requires sustained attention and imagination, engaging the brain in ways that differ from the rapid, fragmented consumption of digital media. This deeper engagement may help strengthen empathy (by inhabiting characters’ perspectives), enhance critical thinking (interpreting themes and morals), and provide a mental slow space to reflect, contrasting with the dopamine-driven scroll cycles of the internet.

### Conclusions

Reading classical fairy tales like Hansel and Gretel and Little Red Riding Hood can indeed serve as a counterbalance to some of the negative effects of internet browsing on young people’s resilience and outlook. Grimms' fairy tales offer timeless lessons in resilience, morality, and hope, qualities that can mitigate the passive consumption, instant gratification, and emotional detachment sometimes exacerbated by internet browsing. Classic Grimms' fairy tales can be ancient little engines of resilience: They spark connection, reflection, and hope.

## Appendix

Multimedia Appendix 1 CONSORT-SPI 2018 Checklist: Reporting Randomized Trials of Social and Psychological Interventions.

## Abbreviations

AVE: average variance extracted
CONSORT-SPI: Consolidated Standards of Reporting Trials statement for social and psychological interventions

## Data Availability

Study data are available from the authors upon reasonable request.
